# Resolving shortages of prescription drugs: the case for public-private collaboration

**DOI:** 10.1186/s13584-017-0152-5

**Published:** 2017-05-10

**Authors:** Eric Efraim Katz

**Affiliations:** Pharma and Healthcare Dimensions, 6001 Stuart Ave., Baltimore, MD 21209 USA

**Keywords:** Pharmaceuticals, Drug shortage, Public-private partnership, FDA, Ministry of Health, Prescription drugs, Collaboration

## Abstract

The recent IJHPR article by Schwartzberg and colleagues presents new data on the growing problem of prescription drug shortages. Resolving shortages typically involves many participants: government, industry, physicians and healthcare facilities. Israel has a strong record of informal collaboration that can fix drug shortages quickly. The success of Israel’s informal collaborations, as well as its formal partnerships, deserves broader recognition at home and more attention from the international community.

## Introduction

The thoughtful IJHPR article by Schwartzberg, et al. [1], offers important contributions to international perspectives on prescription drug shortages (DS).

First, the article offers fresh evidence that shortages damage the cost, quality, and delivery of care. Shortages hurt providers, payers, and most of all, patients. Second, the authors analyze a list of DS causes,^1^ some related to rational business decisions of drugmakers, others generated by regulators as fallout from their primary goal of maintaining a safe drug supply.^2^ The broad spectrum of causes, alone, gives cause for concern.

Finally, just as the pain of drug shortages may have many causes, solutions will range from market-based to clinical and even statutory. The cost of resolving shortages might fall largely on drugmakers, in upgrading plants and expanding production. Other corners of the health care system will also pay a cost, such as providers becoming more prone to medical errors using unfamiliar drugs, or suppliers losing negotiating strength when buying substitute drugs on short notice from a monopolistic seller.

Pulling all these considerations together suggests that effective solutions may require multiple parties working in collaboration. No single perspective – whether from the public or the private sector – can resolve shortages and ensure public health. Typically, the goals of the parties won’t coincide, so the partnership-building process itself creates real value, in its own right, for improving health status and healthcare delivery. The Israeli model of collaborative problem-solving is a clear learning opportunity for officials worldwide.

## The US model for resolving shortages: effective, up to a point

In the US, solutions for serious drug shortages might not always rest on the common-sense collaboration that Israel holds as a value. The Food and Drug Administration (FDA) holds the lead for resolving shortages, under legal authorities that Congress strengthened in 2013.^3^ While the FDA works closely with its regulated industries toward positive results, the Agency’s powers are neither perfect nor comprehensive.

Some US commentators have noted the broad range of possible fixes to US drug shortages.^4^ One school of thought would grant even broader authority to the FDA, including power to move a generic drug application to the front of the review queue to resolve a drug shortage. Another point of view focuses on best practices in the private sector. Clearly, though, Americans look to the FDA to keep the drug supply safe and available.

Other US Agencies play a role in addressing drug shortages, but generally play a far more narrow role than the FDA. The Centers for Disease Control and Prevention (CDC) might notice when a shortage develops for antibiotics for critical infections or biologics such as vaccines and immunoglobulins. Similarly, the Drug Enforcement Administration (DEA) may step in when a narcotic under production quotas goes into shortage. Even so, it might be that aside from the FDA, Agencies finding a shortage might not even know where to turn.

The private sector also plays a role, albeit sometimes more limited, in preventing shortages. One hospital group limits its group purchasing to generic drug suppliers known for quality in the finished drug and the raw ingredients, to minimize the risk of safety-related plant shutdowns that lead to shortages.^5^ Drug companies also seem to pay more attention to how business decisions relate to drug availability, perhaps due to new laws requiring advance reporting of potential shortages.

At first glance, the increasing number of serious shortages may suggest a grim outlook for patient care. The sheer number of factors behind a shortage, and the number of parties needed for a resolution, is hardly good news. Certain novel case studies in the Schwartzberg article, though, may help uncover a path to shrinking the growing problem: enhancing collaboration and public-private partnership.

## The Israeli model and lessons for the international arena

The narrative in the Schwartzberg article describes several Israeli models for addressing shortages. The parties capable of fixing the shortages fall into a few groupings, each holding a different perspective on both cause and fix. Examples of the different points of view include:

1. Drugmakers. A manufacturer’s first obligation is to the financial interests of the shareholders. Patients fall behind that, perhaps followed by other business interests including compliance with government regulations.
2. Payers. The health funds have a responsibility to keep members healthy, but also to do so at an affordable price. Unavailable drugs can lead to higher-priced alternative drugs and perhaps higher costs for overall care.
3. Physicians & providers. A physician’s responsibility is to treat the patient with the most appropriate therapies available: the right drug, at the right time. When the optimal drug goes into shortage, the doctor-patient relationship could suffer even if the treatment succeeds.
4. Patients. It might appear at first that the most important party in the healthcare equation – the patient – has little power to resolve a drug shortage. In fact, though, patients and their families have many options for resolving a shortage, one drug and one patient at a time: using a different pharmacy, taking a less convenient dosage form or resorting to private importation.
5. Government. While other nations might isolate pharmaceutical agencies from other public health initiatives, Israel’s Ministry of Health (MoH) holds authority over both drug regulatory powers and multiple public health programs. This multi-faceted role offers the MoH multiple vantage points and multiple tools to address drug shortages. As a result, Israel’s MoH has potential for flexibility and perspective that might not exist in other national models that isolate each activity.

Beyond its contribution of new data and analysis into the nature of drug shortages, the Schwartzberg article shows fresh examples of the role various players from the public and private sectors can play. The article cites how a simple change in labeling, or an accompanying Dear Health Care Provider letter, can solve the case of a Narrow Therapeutic Index (NTI) drug going into shortage and shrinking the range of available therapies. The resolution required collaboration from the MoH, drugmaker and even wholesale purchasers. The broad reach of the MoH, and the openness of other players to a partnership model, gives Israel a head start toward treating and even preventing dangerous shortages of prescription drugs.

What is it about informal collaboration that succeeds? Clearly, the first step toward success is assembling all key players and achieving transparency in identifying the root cause.^6^ It may be difficult for a drugmaker to speak publicly about why it halted production, but the resolution is different if the plant failed a Good Manufacturing Practices (GMP) inspection compared to cases involving a drop in demand or profitability. Informal collaboration, among parties with very different interests (profit, public health, patient care), can fix shortages and save lives.

One refreshing part of the Schwartzberg perspective is that unlike larger nations with more developed regulatory schemes, Israel may have preserved the ability to resolve multi-party issues with a phone call. The article mentions steps like temporary labeling changes in the face of short-term risk of drug allergy. In other nations, this might require very slow Agency-wide action (in the US), or multiple nation-state discussions (in the EU).

Two aspects of these reports deserve special attention. First is the knowledge the article provides through a primary dataset showing the extent of shortages. Second, and perhaps just as instructive, are examples of creative problem-solving to keep market-based shortages from turning into patient-based adverse events.

## Would Israeli methods hold up in the international arena?

Beyond the scope of the Schwartzberg article, but still of great potential value, is to inquire whether the Israeli collaborative spirit for resolving shortages might apply across national borders. Further study might uncover other openings for life-saving partnerships to resolve shortages. Certainly, though, the Israeli methods and success in resolving drug shortages deserve a stronger spotlight in the international arena.

One area for caution: trade-based solutions. The authors describe part of the armamentarium as including export restrictions or increased imports. This might relieve the domestic shortage, but not the worldwide concern. Where every country faces a similar shortage, import-based regulation is a zero-sum game: what Israel finds from another country may simply exacerbate the other nation’s shortage.

That’s not to suggest that collaboration can’t be successful as an international endeavor. Israel produces drug products difficult to manufacture, or not broadly-sourced, which other nations might find in shortage. Successful public-private partnerships, with the MoH identifying worldwide shortages and Israeli industry working to expand markets, could help an Israeli model contribute toward resolving shortages worldwide.

## Summary

Drug shortages are becoming more serious and numerous each year, across clinical areas. The Schwartzberg article outlines an important piece of research, reporting on both data from new reporting systems and policy implications of those datasets. One lesson is the role a collaborative approach can play in resolving drug shortages. Documenting and expanding the success of Israeli public-private partnerships holds promise for public health, both in Israel and internationally.

## Footnotes

1 Shortages result from any lack of availability, anywhere in the healthcare system. This may stem from actions of government (ordering a plant closure due to lapses in Good Manufacturing Practices), the drugmaker (profitability, or the need for plant capacity to make other products), to procurement-based (failure to order enough product to meet a spike in patient demand. http://healthaffairs.org/blog/2016/03/21/unpacking-drug-price-spikes-generics/

2 FDA recognizes the importance of working with industry on the matter. As one official in FDA’s Drug Shortage program phrased it, “Good agency-industry communication can facilitate resolution of a drug shortage.” https://www.fda.gov/Drugs/DrugSafety/DrugShortages/ucm257746.htm

3 The FDA Safety and Innovation Act authorized regulations in the US Federal Register, published July 8, 2015. 80 FR 38915.

4
http://content.healthaffairs.org/content/35/5/798.abstract, doi: 10.1377/hlthaff.2015.1157. Health Aff May 2016 vol. 35 no. 5 798-804.

5 Health Affairs blog piece, supra note 1 (http://healthaffairs.org/blog/2016/03/21/unpacking-drug-price-spikes-generics/)

6
http://healthaffairs.org/blog/2016/06/14/prescription-drug-shortages-data-limitations-in-an-era-of-big-data/
